# Mentors’ Perspectives on Our Commitments to Mentees

**DOI:** 10.1016/j.jaccas.2021.05.006

**Published:** 2021-08-04

**Authors:** Gina Lundberg, Annabelle Santos Volgman, Toniya Singh

**Affiliations:** a Emory University School of Medicine, Marietta, Georgia, USA; b Rush University Medical Center, Chicago, Illinois, USA; c Saint Louis Heart & Vascular, St Louis, Missouri, USA

**Keywords:** awareness, health care economics, lifestyle

Dear mentee, Cardiology is a challenging and exciting field of medicine, and we share your love and passion for taking care of patients with diseases of the heart, arteries, and valves. We experienced the same awe and wonder of the beating heart and the same desire to help patients with cardiovascular pathology as you do now. You remind us of our first love, the early infatuation of cardiology that has grown and developed into a comfortable passion, like a married couple after many years of marriage. You give us renewed joy in cardiology and make us optimistic about the future of our beloved field.

As women in cardiology, we know the discomfort of being the only woman in the room and sometimes being left out of the room. The isolation of having few role models and fewer mentors. The challenges of being heard, respected, and valued for your skills, knowledge, and ideas. As women in cardiology, it is vital for us to mentor, coach, and support young women and under-represented minorities. We need to encourage medical students and residents to choose cardiology, support the female fellows who have chosen cardiology, and nurture them to be future mentors, supporters, coaches, and eventually leaders. The number of women in cardiology has slowly grown from a handful of women to nearly 25% of cardiology fellows (1). Our female ACC Presidents are increasing in number, with our current ACC President, Dipti Itchhaporia, MD, FACC, and Past President Athena Poppas, MD, MACC.

Mentoring is a sworn duty and obligation from the Hippocratic Oath to "gladly share such knowledge as is mine with those who are to follow." Mentoring is a considerable investment of time, energy, and passion. It is a long-term commitment, much like team coaching and even parenting. Like coaching, mentoring means providing encouragement, seeing hidden potential, and tending growth and development while providing valuable guidance. A good coach also knows when you need feedback, hard truths, and redirection delivered in an objective, noncritical way. Like parenting, being a good mentor puts others' needs central; promotes others; and is selfless, giving, and generous.

Building clinical skills is essential for passing boards and becoming confident cardiologists. Developing people skills and leadership skills are also critical for career success, but are generally not taught in our academic institutions. Therefore, many of these soft skills are caught and not taught. Mentors with good personal skills, empathy, compassion, and listening skills are beyond valuable. Mentoring includes sharing our personal stories, struggles with work-life balance, disappointments, and failures. For women in cardiology, this includes decisions on marriage, pregnancy, infertility, and motherhood, and maintaining relationships with family and friends outside of medicine.

Mentoring has evolved to include building networks and opening doors for opportunities. Providing tools for career success is important, but creating connections and opening doors is golden. Being recommended or nominated for committees, writing opportunities, and research trials may be pivotal in a mentees' career path. Mentors must not hoard their speaking invitations, committee invitations, and leadership offers. Passing along a request for a journal review paper, a lecture, a writing request, or committee seat that would be one more thing on your lengthy curriculum vitae to a bright, eager mentee is the chance that could launch their career to new levels. Expanding opportunities for mentees and assisting them to get a seat at the table is the litmus test of being a good mentor. Seeing their mentees follow their dreams, reach goals, and rise in leadership in their chosen area is the long-term objective of all mentors.

A good mentor is also a promoter. A good mentor helps you write papers that get published, enables you to write grants that get funded, and expands your network. They put your name forward and promote you behind the scenes. Because women are less likely to promote or negotiate for themselves, mentors should empower women to acknowledge their talents, skills, and accomplishments and learn that these are valuable. Mentors can help change the mentees' perception of being competent and assertive for their worth instead of being competitive or aggressive to get ahead.

If you have had a good mentor, you have benefited from a good role model and had a positive experience to inspire you to be a good mentor. Unfortunately, if you did not have a good role model or mentor, strive to be the mentor you never had but always wanted.

Mentoring is best practiced at every stage. Begin mentoring during medical school, residency, and fellowship, and then it will be even easier in your early career. By midcareer, you should have several mentees who are gaining a foothold in their respective areas, and by the late-career, you should have many mentees who are succeeding.

Being a mentee comes with specific responsibilities too. You can find a mentor through e-mail, you could approach someone at a meeting, or you could meet someone through a mutual acquaintance. It is essential to be clear on your expectations. It is a good idea to set up regular appointments. Come to your meetings prepared. Articulate your goals and vision, and think about what you are asking for. It is also essential to agree with your mentor on the definition of success, discuss and agree upon the desired outcome, and then determine methods for achieving the goal(s). Determine the necessary expertise and devise strategies for acquiring the skill and knowledge. Develop a plan and use it. It is also important to remember that 1 mentor cannot supply all of your needs, and you may have to consider multiple mentors for various parts of your life and career.

More cardiologists from diverse backgrounds, such as first-generation doctors, socioeconomically disadvantaged backgrounds, under-represented minorities, LGBT, and women, will provide the future with more role models and mentors that have greater cultural understanding and commitment to these minority groups. And a more diverse workforce in cardiology is part of the strategic plan in ACC and similar medical societies.

This letter is a call to action for all cardiologists, especially female and minority cardiologists, to mentor, coach, and sponsor future cardiologists to build an open, equitable pipeline that ensures progress toward gender equity, racial equity, and long-term sustainability of our field (2). "All cardiologists" means men and women, early career and senior career, as well as academic, private, research, and industry cardiologists.

We all know the old proverb, "If you give a man a fish, you feed him for a day, but if you teach him to fish, you feed him for a lifetime." I think this not only applies to the lifelong value of teaching, but also to the lifelong impact of mentoring. If you mentor even 1 person, and she becomes a successful clinician, researcher, or educator, then you will have contributed to all of the future physicians and patients she has touched. We agree that the growth of the virtual networking platforms can transform mentoring from local activity to international activity, especially in areas where female mentors are scarce. We must commit to mentoring our young women in cardiology, and we must become the mentors, coaches, and sponsors that the future of cardiology necessitates. We encourage young women to join us in cardiology to enhance the lives of our patients with cardiovascular disease by increasing diversity in cardiology; we will help you in your journey.

## Funding Support and Author Disclosures

The authors have reported that they have no relationships relevant to the contents of this paper to disclose.
